# Phosphatidylcholine and phosphatidylethanolamine plasmalogens in lipid loaded human macrophages

**DOI:** 10.1371/journal.pone.0205706

**Published:** 2018-10-11

**Authors:** Stefan Wallner, Evelyn Orsó, Margot Grandl, Tatiana Konovalova, Gerhard Liebisch, Gerd Schmitz

**Affiliations:** Institute of Clinical Chemistry and Laboratory Medicine, Regensburg University Medical Center, Regensburg, Germany; Universita degli Studi di Padova, ITALY

## Abstract

**Background:**

Plasmalogens are either phosphatidylcholine (PC P) or phosphatidylethanolamine (PE P) glycerophospholipids containing a vinyl ether moiety in sn-1-position and an esterified fatty acid in sn-2 position. Multiple functions have been proposed, including reservoir of precursors for inflammatory mediators, modulation of membrane fluidity, and anti-oxidative properties. They could therefore play a role under conditions of metabolic stress. Especially enzymatically modified LDL (eLDL) and oxidatively modified LDL (oxLDL) represent modifications of LDL that are taken up by macrophages in atherosclerotic plaques. The aim of this study was to analyze plasmalogen related effects of eLDL and oxLDL in human monocyte derived macrophages, as well as the effects of HDL_3_ mediated deloading.

**Methods:**

Elutriated monocytes from nine healthy donors were differentiated *in vitro* for four days. Macrophages were then loaded with native LDL, eLDL and oxLDL for 24h and subsequently deloaded with HDL_3_ for another 24h. Lipidomic and transcriptomic profiles were obtained.

**Results:**

Loading of macrophages with eLDL and oxLDL led to a transient but strong elevation of lysophosphatidylcholine (LPC) most likely through direct uptake. Only eLDL induced increased levels of total PC, presumably through an induction of PC synthesis. On the other hand treatment with oxLDL led to a significant increase in PC P. Analysis of individual lipid species showed lipoprotein and saturation specific effects for LPC, PC P and PE P species. Membrane fluidity was decreased by the large amount of FC contained in the lipoproteins, as indicated by a lower PC to FC ratio after lipoprotein loading. In contrast the observed changes in the saturated to mono-unsaturated fatty acid (SFA to MUFA) and saturated to poly-unsaturated fatty acid (SFA to PUFA) ratios in PE P could represent a cellular reaction to counteract this effect by producing more fluid membranes. Transcriptomic analysis showed considerable differences between eLDL and oxLDL treated macrophages. As a common feature of both lipoproteins we detected a strong downregulation of pathways for endogenous lipid synthesis as well as for exogenous lipid uptake. Deloading with HDL_3_ had only minor effects on total lipid class as well as on individual lipid species levels, most of the time not reaching significance. Interestingly treatment with HDL_3_ had no effect on membrane fluidity under these conditions, although incubation with HDL_3_ was partially able to counteract the oxLDL induced transcriptomic effects. To investigate the functional effect of lipoprotein treatment on macrophage polarization we performed surface marker flow cytometry. Under our experimental conditions oxLDL was able to partially shift the surface marker pattern towards a pro-inflammatory M1-like phenotype. This is consistent with the consumption of arachidonic acid containing PE P species in oxLDL treated cells, presumably for the synthesis of inflammatory mediators.

**Summary:**

Our findings provide novel data on the lipoprotein induced, lipidomic and transcriptomic changes in macrophages. This can help us better understand the development of metabolic, inflammatory diseases as well as improve our background knowledge on lipid biomarkers in serum.

## Introduction

The coordinated function of the human immune system is inextricably linked to lipid metabolism. Besides their role as exogenous signaling molecules, lipids also control complex endogenous cellular processes such as phagocytic differentiation [1]. Macrophages as the major phagocytic cell type play a central role in innate immunity. They are of great importance for the pathogenesis of atherosclerosis and insulin resistance in metabolic syndrome patients [2]. Fatty streaks in the aorta and other arterial blood vessels consist to a large extent of macrophage derived foam cells that contain large amounts of cholesterol either in lipid droplets or endolysosomes (phospholipidosis) [3, 4]. These lipid loaded cells play a key role in the persistence of local, low grade inflammation [5]. Macrophage phenotypes are classically characterized as a continuum between two extremes: the pro-inflammatory M1 phenotype with high levels of IL-12 and the anti-inflammatory M2 phenotype secreting cytokines such as IL-10 [6]. This polarization also affects macrophage surface marker expression and chemokine secretion [6].

Cholesterol in atherosclerotic lesions accumulates mainly from low density lipoprotein (LDL) particles that represent the major cholesterol transport vehicle in human plasma. Under disease conditions, LDL particles are degraded locally by hydrolytic enzymes. This enzymatically modified LDL (eLDL) is taken up with a higher efficiency by macrophages [7]. It is recognized and internalized into macrophages by type I and type II phagocytosis. This process is mediated by opsonin receptors for complement (CD11b/CD18, CD11c/CD18), pentraxin- and IgG-binding Fcγ-receptors I-III (CD64, CD32, CD16), and the IgM (Fcμ/IgM receptor)[8–10]. The resulting excess uptake ultimately leads to the formation of foam cells.

Furthermore reaction of LDL particles with free radicals and peroxidases leads to the formation of oxidatively modified LDL (oxLDL). oxLDL is more reactive than native LDL or eLDL and leads to tissue damage and attraction of additional pro-inflammatory cells [11, 12]. In contrast to eLDL, it is preferentially taken up through charge and motif dependent scavenger receptors (e.g. CD36 or LOX1R) and damage-associated molecular pattern molecule (DAMP)-receptor complexes [10]. Clinically plasma levels of oxLDL show a correlation with cardiovascular disease while the administration of antioxidants has been shown to exhibit a protective effect against atherosclerosis [13]. Loading of macrophages with oxLDL induces endolysosomal phospholipidosis [10]. oxLDL as well as eLDL, have been identified *in vivo* in human atherosclerotic vessel walls using specific antibodies [4, 14].

Plasmalogens are a class of mostly phosphatidylcholine (PC P) or phosphatidylethanolamine (PE P) containing phospholipids that encompass a vinyl ether moiety in the sn-1-position of the glycerol backbone and an ester bond in sn-2. They are found in all mammalian cells, comprising about 18% of all phospholipids [15] and 65% of all phosphatidylethanolamine (PE) phospholipids. They are particularly abundant in neurons, cardiac and skeletal muscle, but also occur in sizable amounts in platelets, neutrophils and macrophages [16]. Although plasmalogens have been first described 80 years ago, their physiological purpose even now remains partially enigmatic. Over the years a range of functions has been proposed, that are mostly attributable to the characteristic ether-bond: (1) storage of precursor fatty acids in sn-2 position for the synthesis of n-3 and n-6 prostanoids as inflammatory mediators, (2) modulation of membrane fluidity and regulation of endo- and exocytosis, and (3) anti-oxidative (scavenger) properties that protect the cell under conditions of oxidative stress [17].

Until recently, atherosclerosis research has focused primarily on abundant plasma lipids such as cholesterol and triglycerides. Plasmalogens and especially cellular plasmalogen species have been studied to a considerably lesser extent. Data from hypertensive patients show an ether-lipid deficiency in their plasma [18]. Therefore in the current study we focused on plasmalogens in human primary macrophages following treatment with eLDL and oxLDL. Furthermore there is evidence that ether lipids are able to modulate transcriptional networks. To this end we additionally performed microarray analysis to study effects on gene expression.

## Materials and methods

### Materials

If not otherwise stated all materials were obtained from Sigma (Munich, Germany). Carrier-free macrophage colony-stimulating factor (M-CSF) was obtained from R&D (Wiesbaden, Germany). HPLC grade methanol and chloroform were from Merck (Darmstadt, Germany), plasmalogen standards from Avanti Polar Lipids (Alabaster, AL, USA).

### Isolation of low density lipoprotein (LDL) and high density lipoprotein subfraction 3 (HDL_3_)

Lipoproteins were isolated from fresh, non-lipemic human plasma of healthy donors by a modified sequential preparative ultracentrifugation in KBr gradients (HDL_3_ d = 1.125–1.21 g/ml and native LDL d = 1.019–1.063 g/mL) followed by extensive dialysis and filter sterilization according to published methods [19]. All lipoprotein concentrations mentioned are protein concentrations determined by the Lowry method. Lipoprotein fractions were stored in the presence of 0.5 mmol/l EDTA at 4°C. None of the blood donors had diabetes mellitus or underwent treatment for arterial hypertension.

### Preparation of enzymatically modified and oxidized LDL

eLDL was generated under sterile conditions. To this end native LDL was diluted to 2 mg/ml protein in PBS (w/o Ca^++^, Mg^++^) and 6.6 μg/ml trypsin (Sigma, Germany) as well as 400 μg/ml cholesterylester hydrolase (Seikagaku, Japan) were added. Subsequently the solution was incubated at 37°C for 48 h.

Oxidized LDL was attained by dialyzing purified LDL fractions (1 mg protein/ml) against 5 μM CuSO_4_ for 40 h. The oxidation process was stopped by repeated and extensive dialysis in PBS/EDTA. Afterwards, the oxLDL was sterile filtered and the protein content was determined by the Lowry method. The degree of oxidation was controlled by electrophoresis.

### Blood cell isolation, *in vitro* differentiation and lipid loading of human monocytes

Blood samples were obtained from nine healthy normo-lipidemic volunteers recruited from blood donors with apoE3/E3 phenotype. Informed consent and approval of the Hospital Ethics Committee were obtained (Universitätsklinikum Regensburg, Ethikkommission der medizinischen Fakultät, proposal 08/119). Donors were fully informed of the possible complications and gave their written consent for the procedure. Blood cells were collected by leukapheresis in a Spectra cell separator (Gambro BCT, CO, USA), followed by counterflow centrifugation elutriation as described elsewhere [20]. In brief, cells were elutriated in the following order: platelets, lymphocytes, monocytes and then granulocytes. Aliquots of the different cell fractions were analyzed for cell purity on a BD FACSCanto II flow cytometer (Becton Dickinson, Heidelberg, Germany) using BD FACSDiva Software. Cell numbers were determined on an ADVIA 120 automated cell counter (Siemens Healthcare Diagnostics GmbH, Bad Nauheim, Germany). Phagocytic differentiation of monocytes to macrophages was conducted by culturing monocytes in macrophage serum-free medium (Invitrogen, Germany) at 10^6^ cells/ml in tissue culture plates (6-well flat bottom; Sarstaedt, Germany) in an incubator (5% CO_2_, 37°C) with the addition of recombinant human monocyte-colony stimulating factor (rhM-CSF, 50 ng/ml, R&D Systems, USA).

### Loading and deloading with lipoproteins

After four days of differentiation, macrophages were loaded with LDL preparations for 24 hours followed by harvesting. eLDL was used at 40 μg/ml and copper oxidized LDL (oxLDL) at 80 μg/ml. Deloading of control, eLDL or oxLDL loaded cells was carried out with HDL_3_ at a concentration of 100 μg/mL for further 24 hours. After washing with PBS, the cell pellets were stored at −80°C. Protein concentrations were measured according to Smith et al. [21] using the bicinchoninic acid assay (BCA) Assay from Uptima-Interchim (Montluçon, France) with serial dilutions of bovine serum albumin as standards.

### Flow cytometry

Flow cytometric analysis of surface marker expression was performed on a FACS Canto II flow cytometer (Becton Dickinson) using a five-color setup. Anti CD206 FITC, Anti CD163 PE and Anti CD14 PerCP were bought from Becton-Dickinson. Anti-CCR7 APC was supplied by R&D systems and Anti CD11c PC7 by IOT/Beckman-Coulter.

### Lipid mass spectrometry

Cell pellets were dissolved in 0.2% SDS solution and disrupted by sonication on ice (Soniprep 150, Beun de Ronde, Abcoude, Netherlands). An aliquot corresponding to 100 μg was used for mass spectrometric lipid analysis. Lipid extraction was performed according to the method of Bligh and Dyer [22] in the presence of not naturally occurring lipid species as internal standards. The chloroform phase was dried in a vacuum centrifuge (SpeedVac, ThermoFisher Scientific) and dissolved in 10 mM ammonium acetate in methanol/chloroform (3:1 vol/vol). Samples were analyzed by ESI-MS/MS in positive ion mode after direct flow injection using the analytical setup and data analysis algorithms described previously [23]. A precursor ion scan of *m/z* 184 specific for phosphocholine containing lipids was used for phosphatidylcholine (PC) and lysophosphatidylcholine (LPC) [23, 24]. PE-plasmalogens were quantified according to the principles described by Zemski, Berry, and Murphy [25]. For this purpose fragment ions of m/z 364, 390 and 392 were used for PE P-16:0, PE P-18:1 and PE P-18:0 species, respectively. After identification of relevant lipid species, selected ion monitoring analysis was performed. Free cholesterol was quantified according to the methodology of Liebisch et al. [26]. Glycerophospholipid annotation is generally based on the assumption of even numbered carbon chains only.

### Gene expression analysis

Cells were harvested, washed in PBS, resuspended in buffer RLT and RNA was isolated using the RNeasy Mini Kit (Qiagen) according to the manufacturer´s instructions. Purity and integrity of the RNA were determined on the Agilent 2100 Bioanalyzer with the RNA 6000 Nano LabChip reagent set (Agilent Technologies). RNA was quantified using the Nanodrop ND-1000—UV/Vis Spectrophotometer (PeqLab).

For gene array analysis we used a modified standard Agilent 4x44K microarray (014850) containing 205 free positions (Agilent Technologies). 201 probes were added to these positions, corresponding to 119 genes previously not represented on the array. 300 ng of total RNA were labeled with Cy3 using the Agilent Quick-Amp Labeling Kit—1 color according to the manufacturer´s instructions. cRNA was purified with the RNeasy Mini Kit (Qiagen). cRNA amounts and labeling efficiency were determined on a NanoDrop ND-1000 photometer (PeqLab). The Agilent Gene Expression Hybridization Kit was used for hybridization. Arrays were incubated for 17 hours at 65°C in Agilent SureHyb chambers in a hybridization oven while rotating. Wash steps were performed according to the manufacturer’s instructions. Scanning was done with the Agilent G2565CA Microarray Scanner System. The resulting TIFF files were processed with Agilent Feature Extraction software (10.7). Microarray data are available in the ArrayExpress database (www.ebi.ac.uk/arrayexpress) under accession number E-MTAB-2298.

### Statistical analysis

All data was analyzed in SPSS 20 (IBM). Means were compared by one-way ANOVA followed by post-hoc testing with LSD correction. P-values of less than 0.05 were regarded as statistically significant.

The hypergeometrical score for transcriptional regulation was calculated as previously described according to the method of Kondrakhin et al. [27]. Significantly regulated transcripts (hypergeometrical score >6) were subjected to the PANTHERdb overrepresentation test (Fisher’s exact test with FDR multiple test correction, release 20170413) and annotated with Reactome pathways [28].

## Results

### Total phospholipids

**Fig 1:**
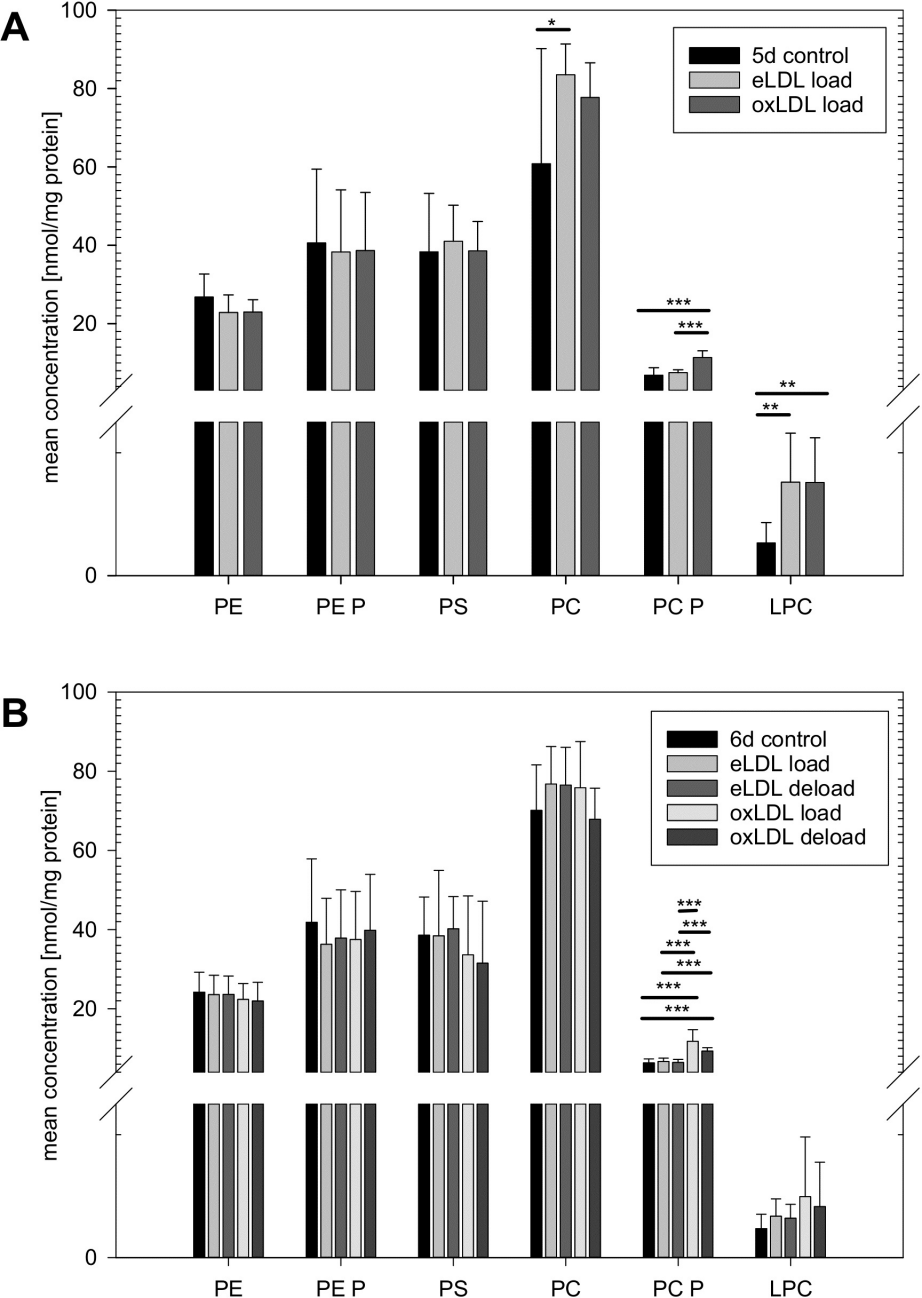
Analysis of total phospholipid levels. (A) Loading of macrophages with modified lipoproteins leads to significant increases in total PC, PC P and LPC species on day 5. (B) Subsequent deloading with HDL_3_ for 24 hours does not have a significant effect compared to loaded control cells. n = 9 for all experiments except n = 8 for 6d MCSF control data, means +/- 1 standard deviation (SD), (* p<0.05, ** p<0.01, *** p<0.001).

Lipid extracts from macrophage cell pellets were analyzed for their phospholipid content after four days of phagocytic differentiation and one additional day of loading with eLDL and oxLDL. As shown in Fig 1A there was no significant effect on total PE, PE P and phosphatidylserine (PS) levels. Loading with oxLDL led to significant increases in PC P and lyso-phosphatidylcholine (LPC) species. The observable increase in total PC levels did not reach statistical significance. Loading with eLDL significantly increased total PC and LPC levels.

To assess the effect of HDL-mediated deloading, cells were incubated for another 24 hours with purified HDL_3_. As shown in Fig 1B, on day six the lipoprotein induced elevations in PC and LPC species were smaller than on day five and partially lost their statistical significance. Particularly levels of LPC species were almost back to control levels. However the significant effect of oxLDL treatment on total PC P persisted also on day six. Treatment with HDL_3_ lead to small reductions of total PC, PC P and LPC in oxLDL loaded cells, but none of these changes was able to reach statistical significance.

### Individual LPC and PC plasmalogen species

**Fig 2:**
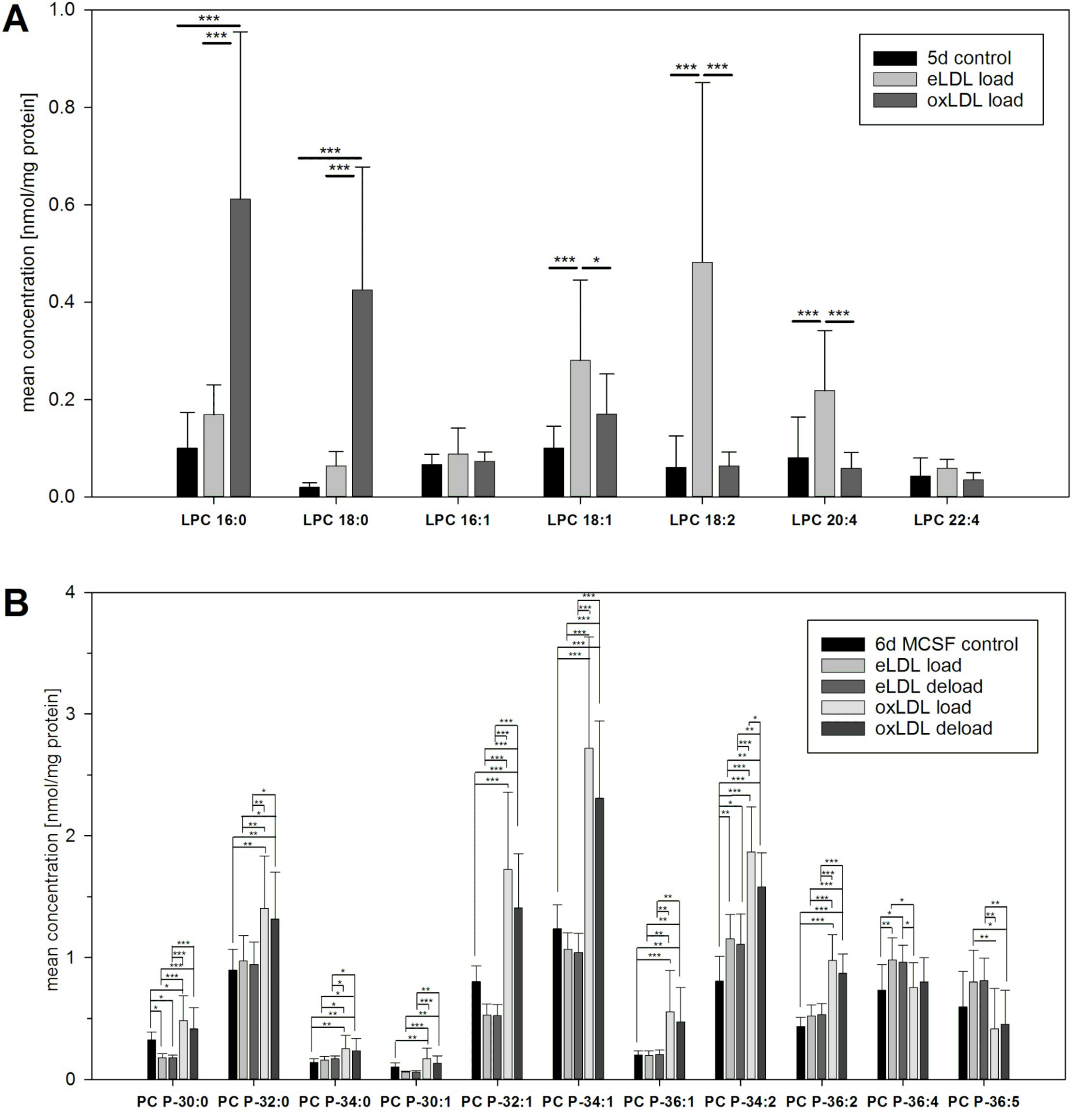
Individual LPC and PC P species after lipid loading. (A) Individual LPC species on day five show saturation and lipoprotein dependent concentration changes (B) Modulation of PC P species after lipoprotein loading and subsequent 24h of HDL_3_ mediated deloading on experimental day six. For **i**ndividual PC P only species with detectable levels are shown. n = 9 for all experiments except n = 8 for 6d MCSF control data., means +/- SD. (* p<0.05, ** p<0.01, *** p<0.001).

Loading with either eLDL or oxLDL resulted in a comparable rise in total LPC on day five (Fig 1). However analysis of individual LPC species exhibited a lipoprotein dependent species pattern (Fig 2A). eLDL primarily raised levels of the unsaturated LPC species 18:1, 18:2 and 20:4. Unfortunately the exact positions of the double bonds could not be inferred from the mass-spectrometric data. Loading with oxLDL on the other hand strongly elevated the saturated LPC species 16:0 and 18:0.

The oxLDL induced increase in total PC P was significant on day five as well as on day six. Therefore the detailed lipid species analysis was performed using measurements from day six. As shown in Fig 2B, especially saturated, mono-unsaturated and di-unsaturated PC P species were prominently higher in oxLDL treated cells compared to untreated controls. Treatment with eLDL lowered PC P-30:0 levels and increased PC P-34:2 and PC P-36:4 levels significantly. In comparison changes induced by eLDL were smaller than those induced by oxLDL.

### Individual PE plasmalogen species

**Fig 3:**
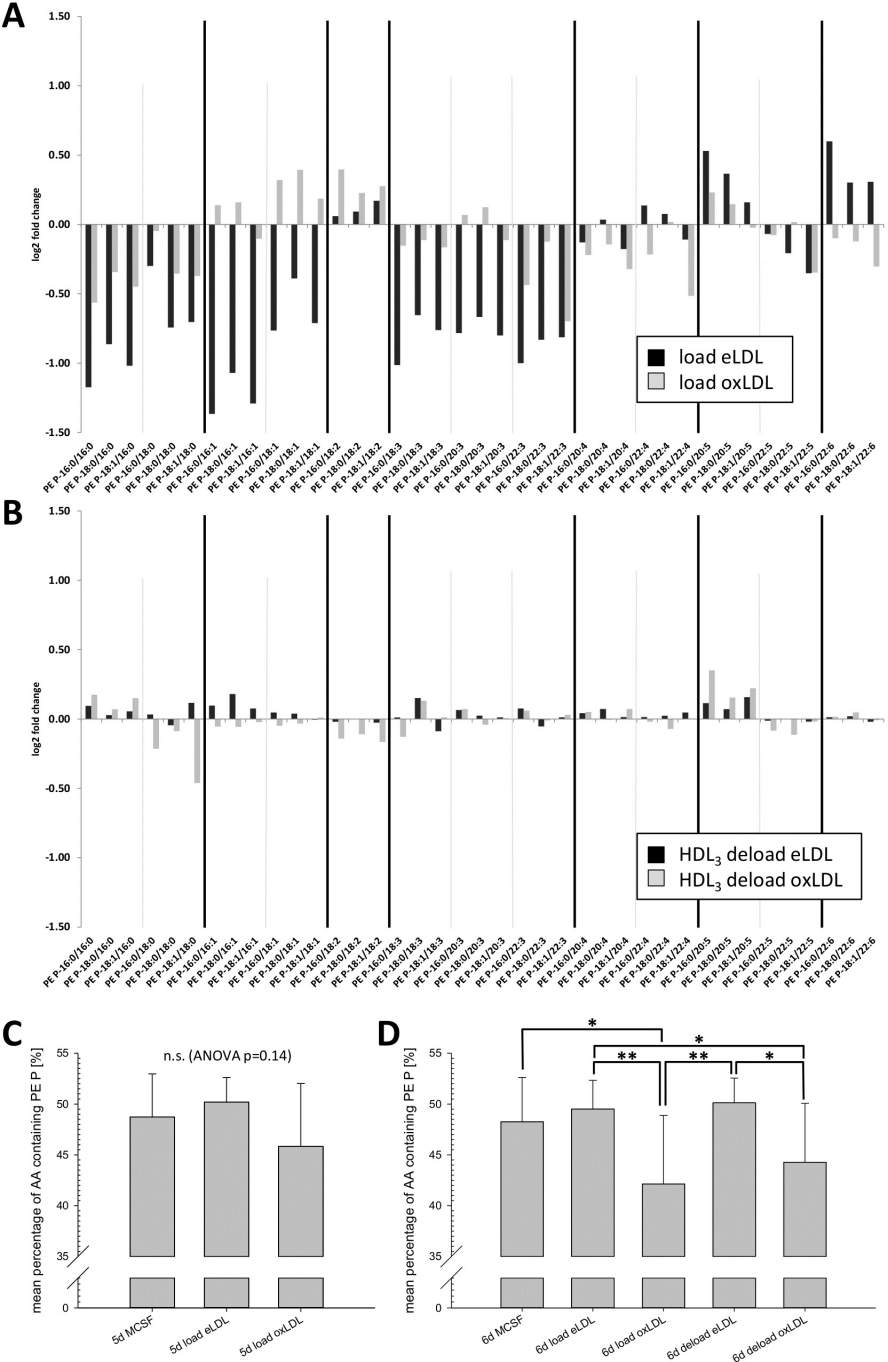
Individual PE plasmalogen species. (A) Relative changes (log2 fold change) of individual plasmalogen species on day five after 24h loading with either eLDL (black bars) or oxLDL (grey bars) in comparison to controls. (B) Relative changes in cells deloaded with HDL_3_ for 24h following eLDL or oxLDL treatment in comparison to loaded controls. (C) Percentage of arachidonic acid (20:4) containing PE Ps relative to total PE Ps 24h after lipoprotein loading. (D) Percentage of arachidonic acid containing plasmalogens 48h after lipoprotein loading and following 24h HDL_3_-mediated deloading in respective cells. n = 9 for all experiments except n = 8 for 6d MCSF control data. C and D: mean percentages +/- SD; (* p<0.05, ** p<0.01).

Lipoproteins contain a variety of PE P species that can be modified by oxidative and enzymatic treatment [29]. Nevertheless as previously shown total PE P levels in macrophages did not change significantly after lipoprotein loading (Fig 1). Instead saturation specific changes could be observed at the level of individual species (Fig 3A and 3B). In eLDL treated cells, all PE P species containing saturated, mono-unsaturated and three-times unsaturated acyl-residues in sn-2 position were lower than in control cells (Fig 3A). PE plasmalogens with 22:6 in sn-2 were increased by eLDL loading. In contrast treatment with oxLDL led to a different and partially diametrical pattern. While sn-2 saturated species were also reduced, albeit to a lower extent, monounsaturated species showed an increase and 22:6 containing PE P species were slightly decreased. Changes induced by loading with oxLDL were in general less pronounced.

Alterations of PE P concentrations induced by HDL_3_ mediated deloading were also considerably smaller than load effects (Fig 3B). Interestingly they also exhibited a saturation dependency with partly opposing effects depending on the loading lipoprotein. Cells that were previously loaded with eLDL showed increases in 16:0, 16:1 and 20:5 containing species. HDL_3_ deloading after oxLDL loading in contrast led to decreases in 18:0, 16:1, 18:1, 18:2 containing species, as well as to increases in 16:0 and 20:5 containing PE Ps.

Arachidonic acid (AA, 20:4) containing plasmalogens represent the lion’s share of all PE P species and account for around 50% of all cellular PE Ps. As shown in Fig 3C and 3D, loading with eLDL does not significantly influence the mean percentage of AA containing PE Ps. oxLDL on the other hand induced a small, but not statistically significant lowering of AA containing plasmalogens 24h after lipid loading that became more pronounced and statistically significant after 48h (Fig 3D, p = 0.011 compared to MSCF control cells). 24h of deloading with HDL_3_ was not able to restore significantly higher levels of AA containing PE Ps (p = 0.35 compared to oxLDL loaded cells).

### Analysis of membrane fluidity

Cellular phospholipid content represents an important factor determining membrane fluidity. Especially the ratios of saturated (SFA) to mono- (MUFA) and poly- unsaturated (PUFA) acyl residue containing lipids as well as the ratio of total PC to free cholesterol ((PC+PC P)/FC) are generally regarded as indicators of membrane fluidity in cellular systems.

**Fig 4:**
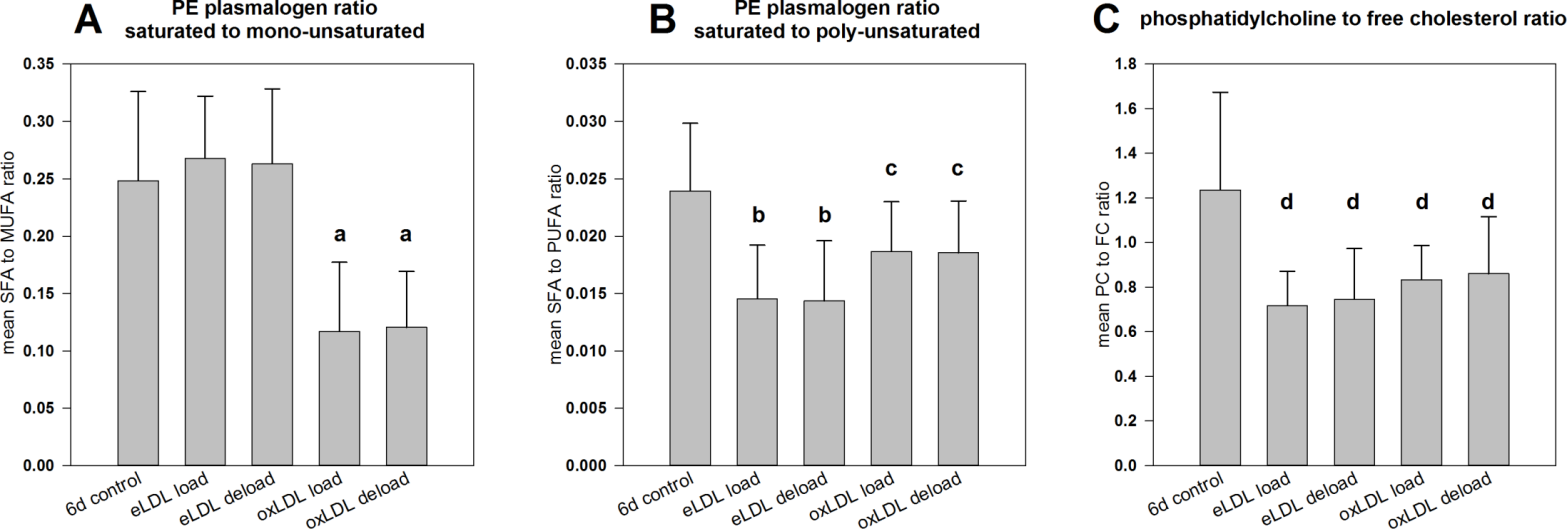
Estimation of membrane fluidity by calculation of lipid ratios on day six. (A) The ratios of saturated to mono-unsaturated PE P species and (B) of saturated to poly-unsaturated PE Ps were calculated as markers for membrane fluidity in control and treated cells on day six. Additionally the mean PC to FC ratio was determined (C). Means +/- SD, n = 9 for control and deload measurements and n = 8 for loaded cells; (a: p<0.001 against control and eLDL loaded cells, b: p<0.001 against control cells, c: p<0.05 against controls, d: p<0.01 against controls).

As shown in Fig 4A, loading with eLDL did not lead to changes in the ratio of SFA to MUFA containing PE P species. However this treatment did significantly lower the SFA to PUFA plasmalogen ratio (Fig 4B). On the other hand loading with oxLDL significantly reduced both, the SFA to MUFA plasmalogen ratio and to a lesser extent also the SFA to PUFA plasmalogen ratio. A lower proportion of plasmalogens containing saturated acyl-residues typically corresponds to more fluid cell membranes. Deloading with HDL_3_ did not have a significant effect on either of these ratios.

The PC to free cholesterol (FC) ratio (Fig 4C) was significantly decreased by treatment with both lipoproteins from 1.7 in macrophages to values around 0.7 to 0.8 in lipid loaded cells. Higher PC to FC ratios in control cells point towards more fluid membranes, while lower ratios in lipoprotein treated cells suggest a more rigid membrane structure. Deloading with HDL_3_ also did not have a significant impact on the PC to FC ratio.

### Transcriptomics

The lipid composition of a cell directly influences a variety of parameters such as membrane fluidity, channel function and clustering of membrane proteins. Still an even wider range of effects can be mediated directly or indirectly by modulation of gene expression. To gain insight into the transcriptomic regulation, we performed Agilent microarrays on control, lipoprotein treated and HDL_3_ deloaded cells. Hypergeometrical scores for the regulation of all detectable transcripts can be found in a supplementary table (S1 File).

**Fig 5:**
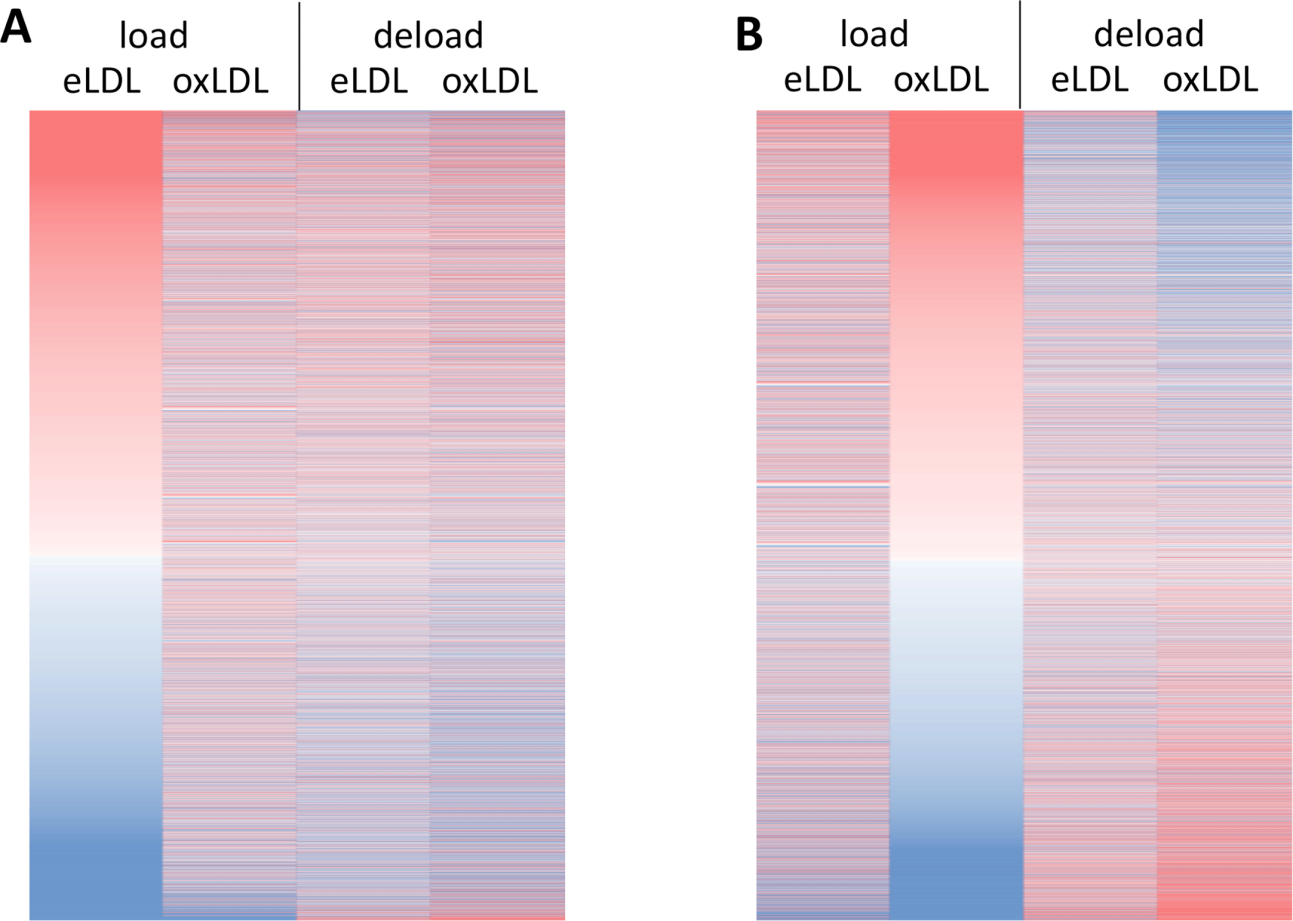
Heatmap of hypergeometrical scores for transcripts quantified in Agilent microarrays. Red signifies positive and blue negative values, indicating up- and down regulation respectively. (A) sorted by “eLDL load” hypergeometrical score, (B) sorted by “oxLDL load” hypergeometrical score. n = 9 microarray profiles from independent donors.

Fig 5 displays a heatmap of gene wise calculated hypergeometrical scores, sorted in descending order in eLDL loaded cells (A) and in oxLDL loaded cells (B). eLDL and oxLDL induced unique patterns of transcriptomic regulation that were considerably dissimilar from each other. As a common feature, both lipoproteins showed downregulation of the same transcripts at the bottom of Fig 5A and 5B. Among others especially transcripts for lipid metabolism related proteins such as the LDL receptor (LDLR), insulin induced gene 1 (INSIG1), fatty acid synthase (FASN), and mevalonate diphosphate decarboxylase (MVD), but also key transcription factors such as Sterol regulatory element-binding transcription factor 1 (SREBF1) and lipid transporters such as ATP Binding Cassette Subfamily A Member 6 (ABCA6) can be found in this group.

Another feature visible in Fig 5A is that deloading of eLDL treated cells with HDL_3_ was barely able to reverse the effects on transcript levels induced by lipid loading. In contrast, deloading of cells loaded with oxLDL (Fig 5B) induced a marked antagonistic effect on previously regulated transcripts. Lipid metabolism related transcripts and chemokine receptors such as GPCR coupled receptors that had been suppressed by loading with oxLDL are strongly upregulated after deloading with oxLDL. On the other hand previously upregulated transcripts related to metallothioneins and amino acid metabolism become strongly downregulated.

**Fig 6:**
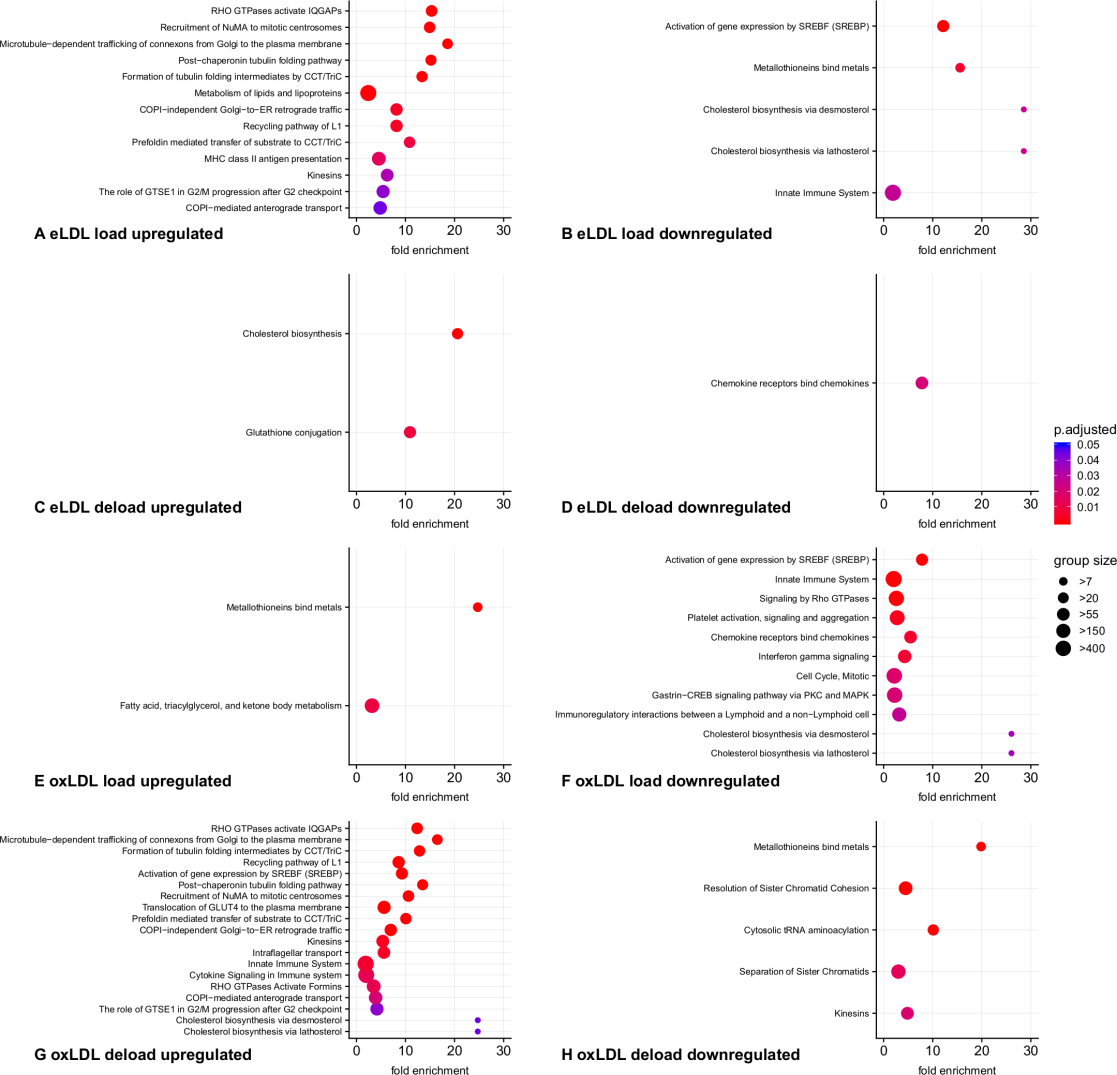
Graphical representation of overrepresented Reactome pathways. For improved clarity only the lowest level of hierarchy in each affected pathway is depicted. Pathways were sorted and color coded according to Bonferroni corrected p-values, dot sizes correspond to Reactome category sizes. n = 9 analyses from independent donors.

To further characterize the transcriptomic effects induced by lipid loading and deloading we used the PANTHER Overrepresentation Test (release 20170413) and annotated the results with Reactome pathways. A cut-off value of >6 was chosen for the hypergeometrical score to classify significantly up- and downregulated transcripts. Resulting, significantly affected pathways are depicted with the respective lowest levels of hierarchy in Fig 6 and additionally listed with full details in the supplement (S2 File). As indicated by the strong enrichment, loading with eLDL suppressed the pathways for cholesterol biosynthesis both via lathosterol and via desmosterol. The pattern of gene regulation was consistent with a sterol regulatory element binding protein (SREBP) mediated mechanism, since known SREBP2 target genes were significantly overrepresented. The LDL-receptor, responsible for the uptake of exogenous cholesterol, was downregulated with the highest hypergeometrical score of all genes investigated. Additionally key enzymes in fatty acid and cholesterol metabolism such as stearoyl-CoA desaturase-1 (SCD) and squalene epoxidase (SQLE) were strongly suppressed. Furthermore expression levels of alpha- and beta-tubulins were increased after lipoprotein loading. Because tubulins play an important role in a variety of pathways such as transport of connexin hemichannels, multiple pathways containing tubulins were also identified by the algorithm. Deloading of eLDL treated cells with HDL_3_ led to a counter-regulatory activation of cholesterol biosynthesis. Furthermore transcripts involved in glutathione metabolism via glutathione S-transferases were upregulated. Following loading with eLDL and also after loading with oxLDL we observed a downregulation of C-C and C-X-C chemokine receptors and ligands.

Similar to treatment with eLDL, loading with oxidized LDL impeded endogenous lipid synthesis and external lipid uptake in a SREBP dependent manner. Furthermore lipid catabolism was induced through short-chain acyl-CoA dehydrogenase (ACADS), acyl-CoA synthetase family member 2 (ACSF2) as well as the lipid droplet forming protein perilipin 2. Interestingly transcripts for HLA class II molecules and genes involved in interferon-γ signaling were also diminished. Potentially in response to copper oxidized LDL, metallothioneins showed increases in transcript levels.

Deloading of oxLDL treated macrophages led to the upregulation of transcripts responsible for cholesterol biosynthesis via lathosterol as well as desmosterol. SREBP2 targets were found statistically enriched. Likewise we observed potential Rho GTPase mediated changes in cytoskeleton associated transcripts. A wide range of tubulin and actin mRNAs were upregulated. Upregulated genes were also found in pathways involved in cytokine signaling and the innate immune system. Especially C-C motif chemokines were overrepresented in the microarray dataset. A downregulation was induced by HDL_3_ in the categories metallothionin binding proteins, cytosolic tRNA aminoacylation, kinesins, and transcripts related to mitosis and meiosis.

### M1/M2 phenotype flow cytometry

**Fig 7:**
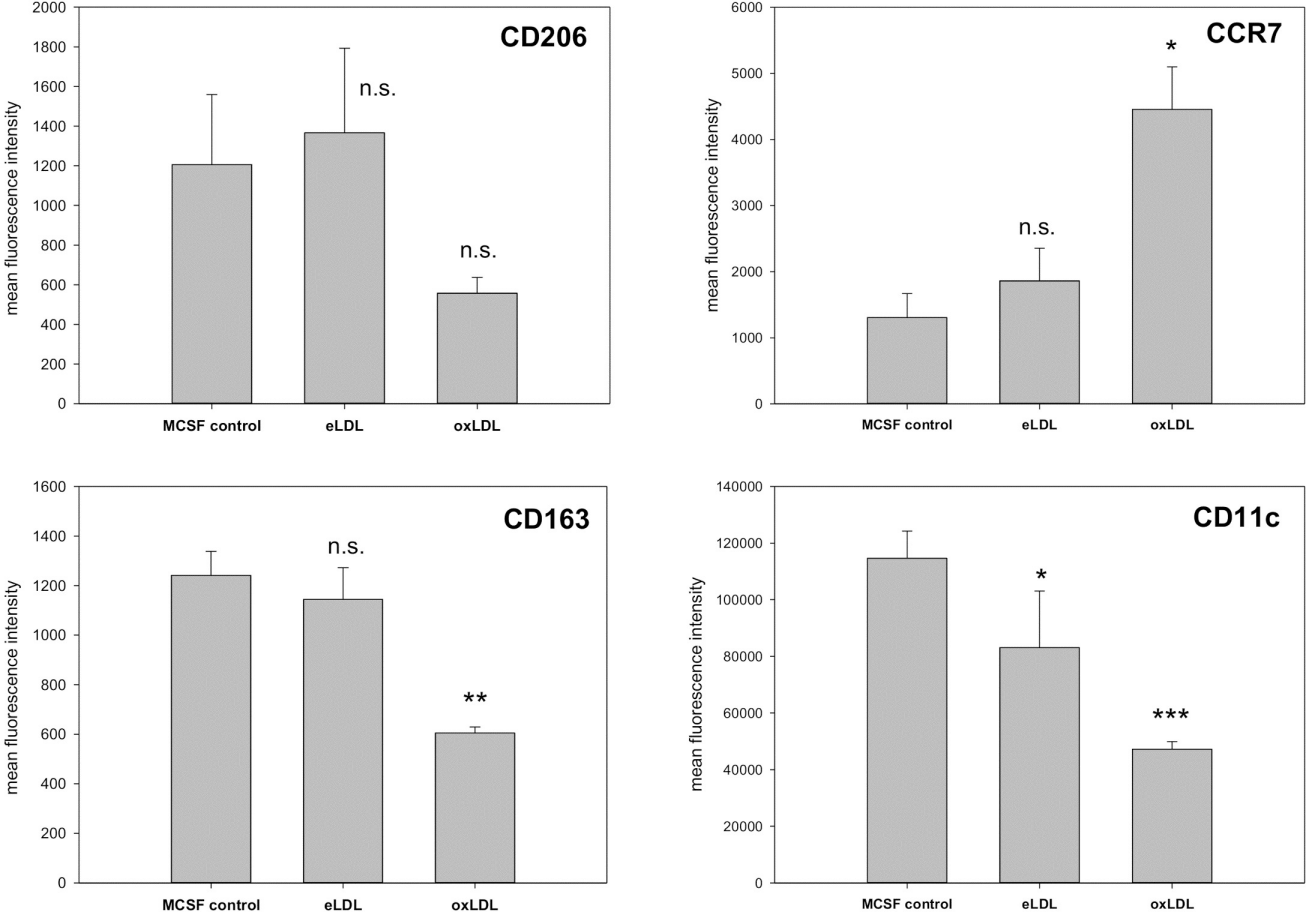
Flow cytometric analysis of M1/M2 surface markers in lipoprotein-treated macrophages. CD163 (scavenger receptor cysteine-rich type 1 protein M130; hemoglobin/haptoglobin receptor) and CD206 (C-type 1 lectin receptor; mannose receptor) were used as M2 markers while CCR7 (CD197) and CD11c served as M1 markers. Loading with eLDL only induced a statistically significant decrease in CD11c surface expression. On the other hand loading with oxLDL led to significant decreases in CD163 and CD11c, as well as to increases in CCR7 and a not statistically significant decrease in CD206. n = 6, means +/- SD; (*p<0.05, ** p<0.01, *** p<0.001).

Depending on the microenvironment macrophages are able to develop either a classically activated M1 phenotype or an alternatively activated M2 phenotype. While M1 macrophages stimulate inflammation, M2 macrophages curb inflammation and play an important role in tissue repair processes [6, 30]. Therefore we hypothesized that exposure to eLDL or oxLDL might modulate macrophage polarization. CD163 (scavenger receptor cysteine-rich type 1 protein M130; hemoglobin/haptoglobin receptor) and CD206 (C-type 1 lectin receptor; mannose receptor) were used as M2 markers while CCR7 (C-C chemokine receptor type 7, CD197) and CD11c served as M1 markers. As shown in Fig 7 treatment with eLDL did only reduce CD11c surface expression significantly. All three other markers remained unaffected, indicating only a minor influence on the M1/M2 phenotype. On the other hand oxidized LDL led to lower expression of CD206 as well as of CD163 and CD11c, while leading to higher levels of CCR7. Therefore three of the four marker proteins are consistent with a shift towards an M1 phenotype after treatment with oxLDL.

## Discussion

Clearance of lipids and lipoproteins by macrophages is known to play a central role in the biology of cardiovascular disease. Enzymatic and oxidative modifications are the most common ways of lipoprotein modification and *in vivo* a combination of both contributes to the formation of atherosclerotic plaques [31]. For the current study we used a model system with eLDL and oxLDL to study their effects on human monocyte-derived macrophages individually.

When macrophages are exposed to lipids, there are multiple ways how this can influence the cell. First of all lipoprotein derived lipids can be directly incorporated into cell membranes and change their structure and physiological properties. The lipid composition of eLDL and oxLDL has been reported in a previous publication by our group [29]. Both lipoproteins are very rich in cholesteryl esters, as well as in free cholesterol. To a lower degree they contain PC and LPC. PE and phosphatidylinositol (PI) species are only present in relatively small amounts. In direct comparison eLDL encompasses a higher amount of free cholesterol than oxLDL, while oxLDL contains relatively higher quantities of PC and LPC species.

In our experiments *in vitro* loading of macrophages induced specific alterations in cellular phospholipid levels that did transiently and partially correlate with the lipoprotein lipid pattern. On day five total PE and PE P levels remained stable, while total PC, PC P and especially LPC levels were increased significantly. This indicates an instant lipid uptake from the lipoprotein. On day six most of the changes from day five were not detectable any more. Only the elevation of LPC after oxLDL loading remained statistically significant. Therefore cellular lipid metabolism seems to be able to swiftly cope with the exogenous lipid load and preserve the global lipid composition of the cell. Nevertheless chronic exposition of macrophages to modified lipoproteins in an atherosclerotic lesion could lead to chronically elevated levels of LPC and other phospholipids in these cells. This corresponds well with observations describing raised levels of oleic acid (18:1 in sn-2) containing LPC species as novel biomarkers for the atherogenic state [32].

Furthermore in fibroblasts free cholesterol is known to be able to upregulate PC biosynthesis [33]. In our lipoprotein treated macrophages we potentially observed a similar effect. Despite a lower content of PC in eLDL particles, total PC levels increased stronger after loading with eLDL compared to loading with oxLDL. Moreover PE synthesis can be inhibited in fibroblasts by incubation with free cholesterol. In our experiments we only observed visibly but not statistically significantly lower PE levels after lipoprotein loading. We did however detect a shift toward polyunsaturated PE plasmalogen species through a reduction of saturated and monounsaturated species.

Surprisingly HDL_3_ mediated deloading only had a very limited influence on the cellular lipidomic profile. Day six macrophages were able to normalize levels of most lipid species already without lipoprotein mediated deloading. Only PC P-34:2 was found significantly lower after deloading with HDL_3_.

The fluidity of the membrane bilayer represents a crucial factor influencing membrane dependent functions such as migration, mitosis, phagocytosis and vesicular trafficking. Among others the PC to FC ratio is generally regarded as a marker of membrane fluidity. In our experiments as well as in atherosclerotic plaques, cells are exposed to abundant amounts of free cholesterol. Correspondingly we detected a lower total PC to FC ratio after lipoprotein treatment. The PC to FC ratio was decreased by treatment with both lipoprotein fractions from 1.7 in control macrophages to values around 0.6 in lipid loaded cells. A previous study on primary human blood cells yielded values of 1.9 in monocytes, 1.3 in lymphocytes, 1.1 in granulocytes, 0.8 in platelets and 0.3 in red blood cells [16]. Higher ratios indicate more fluid membranes that enable cells to migrate more easily and that facilitate phagocytic activity and vesicular processing [34, 35]. Exposure of macrophages to high amounts of free cholesterol therefore seems to lead to less fluid membranes that are more susceptible to mechanical damage [36, 37]. Interestingly the PE P saturation pattern did show opposing effects that could be able to counteract the increased membrane rigidity induced by the high FC load. Plasmalogens are regulators of membrane fluidity and affect membrane dynamics through their ether bond [38]. While saturated and monounsaturated species in sn-2 position lead to more stable structures, plasmalogens with higher degrees of unsaturation result in more fluid membranes. Indeed the relative amount of saturated PE P species decreased after lipid loading with either lipoprotein in comparison to mono-unsaturated and poly-unsaturated PE P species. These findings are also consistent with a previous NMR study that showed a significant shift in the degree of saturation towards mainly polyunsaturated fatty acid chains in the mobile lipid pool of eLDL loaded macrophages [39]. Interestingly deloading with HDL_3_ did not have an effect on the analyzed membrane fluidity marker ratios.

Lipid raft microdomains are closely connected to membrane fluidity. They are cholesterol-rich and harbor a wide range of signaling molecules [40, 41]. They therefore play crucial roles in cell-to-cell signaling, survival, immune-receptor signaling as well as in endocytosis [42]. The proteins within lipid rafts are sensitive to changes in the lipid composition and rising or falling levels of lipids can activate associated proteins such as death receptors and influence apoptosis [43]. Plasmalogens represent a major constituent of lipid raft microdomains and are able to enhance raft stability [44]. The observed decrease in saturated and monounsaturated PE Ps in eLDL treated cells might therefore lead to more fluid and less stable lipid raft microdomains and subsequently facilitate signal transduction processes. Interestingly plasmalogen deficient mice exhibit disrupted lipid raft formation [41].

To examine the effects of lipid loading on gene expression we performed Agilent microarray analysis. Treatment with eLDL as well as with oxLDL led to a strong downregulation of endogenous cholesterol biosynthesis and external uptake. As indicated by Panther pathway overrepresentation analysis this is likely mediated through sterol regulatory element binding proteins (SREBPs). SREBPs represent a family of transcription-factors that play key roles in the regulation of sterol metabolism. Especially SREBP-2 is responsible for the feedback regulation of *de-novo* cholesterol biosynthesis and known to control key transcripts [45]. Also cholesterol overloading is known to lead to the activation of the liver X receptor (LXR) [46]. In addition levels of PUFA containing PE Ps play a direct role in the regulation of cellular cholesterol via a concentration-dependent increase in sterol-O-acyltransferase-1 (SOAT1) [47]. These effects are generally more pronounced in eLDL loaded cells. While cholesterol taken up from oxLDL is trapped in endolysosomes and leads to phospholipidosis, free cholesterol taken up from eLDL accumulates in the ER and leads to rapid lipid droplet formation [10]. Interestingly deloading with HDL_3_ was able to partially rescue the transcriptomic expression profile only in oxLDL treated cells. The effects in eLDL loaded cells were substantially smaller. Therefore high concentrations of HDL cholesterol *in vivo* could be able to transcriptomically attenuate the pro-inflammatory effects of oxidized lipoprotein components on macrophages.

To investigate if lipoprotein loading is able to influence the phenotype of macrophages we used a flow cytometric approach. Using two typical M1 and two typical M2 surface markers we found a shift towards a more M1-like phenotype after oxLDL treatment. Three of the four markers were changed towards the M1 phenotype. Functionally this points towards a higher inflammatory potential. Further evidence of an increased inflammatory potential could be observed in the lipidomic analysis of arachidonic acid containing PE plasmalogens. These abundant species serve as a reservoir for the synthesis of prostanoids and other inflammatory mediator molecules. Potentially due to the consumption in downstream synthesis processes, oxLDL loaded macrophages showed a significantly lower content of PE P with arachidonic acid in sn-2 than control or eLDL loaded cells.

These findings are also interesting in light of the transcriptomic effect we found on transcripts associated with the Reactome category microtubules. Microtubules are for example used for the secretion of MMP-9 in macrophages [48]. Also assembly of the NLRP3 inflammasome is mediated by alpha tubulin [49]. A Rho-GTPase mediated modulation of the microtubule system could therefore also alter the inflammatory potential of lipid exposed macrophages. Intracellular accumulation of fatty acids, especially stearic acid has been previously associated with development of an pro-inflammatory M1 phenotype in macrophages [50].

Unfortunately only limited data are available about the associations of plasmalogens with human diseases. However there is increasing evidence that plasmalogens are significantly involved in chronic metabolic disorders. In general lower plasmalogen levels have been linked to disease development and progression. For example decreased plasmalogen levels in serum are associated with hypertension, obesity, coronary artery disease, and myocardial infarction [18, 51–54]. This also seems to be a metabolic rather than a genetic effect since obese twins exhibit lower serum plasmalogens than their non-obese healthy siblings [51]. Conversely aerobic training and a healthy dietary intervention lead to increased serum plasmalogen levels [55, 56]. Likewise plasmalogen supplementation was able to attenuate atherosclerosis in a mouse model [57].

In human macrophages we found no significant changes of total PE P levels after lipoprotein loading. However there was a clear saturation dependent modulation of the plasmalogen species pattern. Especially eLDL induced isolated reductions in saturated, mono- and di-unsaturated species. Furthermore loading with eLDL did significantly decrease PC P-30:0 and significantly increase PC P-34:2 and PC P-36:4. In contrast loading with oxLDL did significantly increase total PC P levels. Therefore the observed short-term effects in macrophages seem to diverge from long-term observations in plasma. Beyond that our data also show diminished levels of linolenic acid (18:3) containing plasmalogens after eLDL loading. The n-3 fatty acid alpha-linolenic acid (18:3 n-3) possesses a range of beneficial effects in cardiovascular disease [58]. In our mass spectrometric setup it is not possible to differentiate between alpha- and gamma-linolenic acid as plasmalogen residues. Therefore a loss of the beneficial effects of alpha linolenic acid (18:3 n-3) is speculative at the moment.

Decreased serum ether lipids and increased LPC species represent some of the earliest markers for the development of type 1 diabetes (T1D) in children. These changes are present even before autoantibodies are detectable, suggesting metabolic dysregulation even preceding the immune response [59]. Still if the altered lipid pattern is causative for the development of the disease or if dyslipidemia is a secondary metabolic effect remains yet unclear. Anti-oxidative properties might form the link between low plasmalogen levels and the development of T1D. Beta cells only possess a very low anti-oxidative capacity [60]. Therefore lower total plasmalogen levels might render beta-cells more susceptible to metabolic stress and damage.

Furthermore PE, PI and phosphatidylglycerol (PG), but not PC species show positive associations with type 2 diabetes and prediabetes. Here it has also been speculated that increased levels of PE P as a reservoir for arachidonic acid could contribute to increased synthesis of proinflammatory eicosanoids [61]. This is consistent with our findings of significantly lower relative amounts of AA containing PE Ps after oxLDL loading, as well as with our flow cytometric findings. Furthermore eLDL loading led to lower cellular levels of 20:3 DGLA containing species that could serve as precursors for prostaglandin E1 (PGE1) and prostaglandin F1 (PGF1) production.

## Summary and conclusion

Treatment of human macrophages with eLDL and oxLDL induced transient changes in cellular phosopholipid levels. Some of these effects, particularly in PC and PE plasmalogens were saturation dependent and could not be fully explained by direct lipid uptake. Intracellularly they were accompanied by the downregulation of endogenous cholesterol synthesis and exogenous uptake, as well as potentially an induction of PC synthesis and an inhibition of PE synthesis. Very likely these adaptions serve the purpose of keeping the lipidomic composition of the cell and its organelles as stable as possible, as we examined for membrane fluidity and lipid raft function. Functionally the macrophages exhibited a shift towards a more M1 like marker pattern after lipid loading. This finding is consistent with lipidomics showing the consumption of arachidonic acid containing PE plasmalogens and most likely indicates the induction of a pro-inflammatory phenotype. Our data therefore provide some novel information for the understanding of lipid biomarkers that have been described before in serum.

## Supporting information

S1 File Modulation of gene expression in lipoprotein treated macrophages. Hypergeometrical scores were calculated for each lipoprotein treatment versus untreated control cells as well as for HDL_3_ deloaded cells in comparison to loaded controls. A hypergeometrical score of >6 can been regarded as statistically significant.
(XLSX)

S2 File PANTHER overrepresentation test results, annotated with Reactome pathways. Regulated transcripts with a hypergeometrical score (HG) of more than six were analyzed for overrepresentation in Reactome pathways using the PANTHER engine. Resulting p-values were corrected for multiple testing. Pathways are listed in tables separated by lipoprotein (eLDL and oxLDL), direction of change (up- and downregulated) and treatment (loading and deloading).
(DOCX)
