# Supplementary material for: Phosphatidylcholine and phosphatidylethanolamine plasmalogens in lipid loaded human macrophages
Source: PLoS One. 2018 Oct 11;13(10):e0205706. doi: 10.1371/journal.pone.0205706 (PMC6181407; doi:10.1371/journal.pone.0205706)
Supplement: S2 File — Regulated transcripts with a hypergeometrical score (HG) of more than six were analyzed for overrepresentation in Reactome pathways using the PANTHER engine. Resulting p-values were corrected for multiple testing. Pathways are listed in tables separated by lipoprotein (eLDL and oxLDL), direction of change (up- and downregulated) and treatment (loading and deloading). (DOCX) [file pone.0205706.s002.docx]

**Upregulated by eLDL loading (HG>6)**

|  | [**#**](http://www.pantherdb.org/tools/compareToRefList.jsp?sortOrder=2&sortList=Homo%20sapiens) **total** | [**#**](http://www.pantherdb.org/tools/compareToRefList.jsp?sortOrder=2&sortList=Client%20Text%20Box%20Input&sortField=num) **found** | [**expected**](http://www.pantherdb.org/tools/compareToRefList.jsp?sortOrder=2&sortList=Client%20Text%20Box%20Input&sortField=exp) | [**Fold Enrichment**](http://www.pantherdb.org/tools/compareToRefList.jsp?sortOrder=2&sortList=Client%20Text%20Box%20Input&sortField=foldEnrich) | [**P value**](http://www.pantherdb.org/tools/compareToRefList.jsp?sortOrder=1&sortList=Client%20Text%20Box%20Input&sortField=pval) |
| --- | --- | --- | --- | --- | --- |
| [Microtubule-dependent trafficking of connexons from Golgi to the plasma membrane](http://www.reactome.org/PathwayBrowser/#/R-HSA-190840) | [18](http://www.pantherdb.org/tools/gxIdsList.do?acc=R-HSA-190840&reflist=1) | [8](http://www.pantherdb.org/tools/gxIdsList.do?acc=R-HSA-190840&list=Client%20Text%20Box%20Input&organism=Homo%20sapiens) | .43 | 18.57 | 3.39E-05 |
| ↳ Transport of connexons to the plasma membrane | [19](http://www.pantherdb.org/tools/gxIdsList.do?acc=R-HSA-190872&reflist=1) | [9](http://www.pantherdb.org/tools/gxIdsList.do?acc=R-HSA-190872&list=Client%20Text%20Box%20Input&organism=Homo%20sapiens) | .45 | 19.79 | 2.54E-06 |
| ↳ Gap junction assembly | [36](http://www.pantherdb.org/tools/gxIdsList.do?acc=R-HSA-190861&reflist=1) | [9](http://www.pantherdb.org/tools/gxIdsList.do?acc=R-HSA-190861&list=Client%20Text%20Box%20Input&organism=Homo%20sapiens) | .86 | 10.44 | 5.58E-04 |
| ↳ Gap junction trafficking | [47](http://www.pantherdb.org/tools/gxIdsList.do?acc=R-HSA-190828&reflist=1) | [9](http://www.pantherdb.org/tools/gxIdsList.do?acc=R-HSA-190828&list=Client%20Text%20Box%20Input&organism=Homo%20sapiens) | 1.13 | 8.00 | 4.88E-03 |
| ↳ Gap junction trafficking and regulation | [48](http://www.pantherdb.org/tools/gxIdsList.do?acc=R-HSA-157858&reflist=1) | [9](http://www.pantherdb.org/tools/gxIdsList.do?acc=R-HSA-157858&list=Client%20Text%20Box%20Input&organism=Homo%20sapiens) | 1.15 | 7.83 | 5.78E-03 |
| [RHO GTPases activate IQGAPs](http://www.reactome.org/PathwayBrowser/#/R-HSA-5626467) | [30](http://www.pantherdb.org/tools/gxIdsList.do?acc=R-HSA-5626467&reflist=1) | [11](http://www.pantherdb.org/tools/gxIdsList.do?acc=R-HSA-5626467&list=Client%20Text%20Box%20Input&organism=Homo%20sapiens) | .72 | 15.32 | 5.48E-07 |
| [Post-chaperonin tubulin folding pathway](http://www.reactome.org/PathwayBrowser/#/R-HSA-389977) | [22](http://www.pantherdb.org/tools/gxIdsList.do?acc=R-HSA-389977&reflist=1) | [8](http://www.pantherdb.org/tools/gxIdsList.do?acc=R-HSA-389977&list=Client%20Text%20Box%20Input&organism=Homo%20sapiens) | .53 | 15.19 | 1.55E-04 |
| Recruitment of NuMA to mitotic centrosomes | [28](http://www.pantherdb.org/tools/gxIdsList.do?acc=R-HSA-380320&reflist=1) | [10](http://www.pantherdb.org/tools/gxIdsList.do?acc=R-HSA-380320&list=Client%20Text%20Box%20Input&organism=Homo%20sapiens) | .67 | 14.92 | 4.50E-06 |
| ↳ Recruitment of mitotic centrosome proteins and complexes | [91](http://www.pantherdb.org/tools/gxIdsList.do?acc=R-HSA-380270&reflist=1) | [15](http://www.pantherdb.org/tools/gxIdsList.do?acc=R-HSA-380270&list=Client%20Text%20Box%20Input&organism=Homo%20sapiens) | 2.18 | 6.89 | 1.79E-05 |
| ↳ Centrosome maturation | [91](http://www.pantherdb.org/tools/gxIdsList.do?acc=R-HSA-380287&reflist=1) | [15](http://www.pantherdb.org/tools/gxIdsList.do?acc=R-HSA-380287&list=Client%20Text%20Box%20Input&organism=Homo%20sapiens) | 2.18 | 6.89 | 1.79E-05 |
| ↳ G2/M Transition | [185](http://www.pantherdb.org/tools/gxIdsList.do?acc=R-HSA-69275&reflist=1) | [18](http://www.pantherdb.org/tools/gxIdsList.do?acc=R-HSA-69275&list=Client%20Text%20Box%20Input&organism=Homo%20sapiens) | 4.43 | 4.06 | 1.54E-03 |
| ↳ Mitotic G2-G2/M phases | [187](http://www.pantherdb.org/tools/gxIdsList.do?acc=R-HSA-453274&reflist=1) | [18](http://www.pantherdb.org/tools/gxIdsList.do?acc=R-HSA-453274&list=Client%20Text%20Box%20Input&organism=Homo%20sapiens) | 4.48 | 4.02 | 1.79E-03 |
| [Formation of tubulin folding intermediates by CCT/TriC](http://www.reactome.org/PathwayBrowser/#/R-HSA-389960) | [25](http://www.pantherdb.org/tools/gxIdsList.do?acc=R-HSA-389960&reflist=1) | [8](http://www.pantherdb.org/tools/gxIdsList.do?acc=R-HSA-389960&list=Client%20Text%20Box%20Input&organism=Homo%20sapiens) | .60 | 13.37 | 4.06E-04 |
| ↳ Cooperation of Prefoldin and TriC/CCT in actin and tubulin folding | [32](http://www.pantherdb.org/tools/gxIdsList.do?acc=R-HSA-389958&reflist=1) | [8](http://www.pantherdb.org/tools/gxIdsList.do?acc=R-HSA-389958&list=Client%20Text%20Box%20Input&organism=Homo%20sapiens) | .77 | 10.44 | 2.53E-03 |
| [Prefoldin mediated transfer of substrate to CCT/TriC](http://www.reactome.org/PathwayBrowser/#/R-HSA-389957) | [27](http://www.pantherdb.org/tools/gxIdsList.do?acc=R-HSA-389957&reflist=1) | [7](http://www.pantherdb.org/tools/gxIdsList.do?acc=R-HSA-389957&list=Client%20Text%20Box%20Input&organism=Homo%20sapiens) | .65 | 10.83 | 9.13E-03 |
| Recycling pathway of L1 | [46](http://www.pantherdb.org/tools/gxIdsList.do?acc=R-HSA-437239&reflist=1) | [9](http://www.pantherdb.org/tools/gxIdsList.do?acc=R-HSA-437239&list=Client%20Text%20Box%20Input&organism=Homo%20sapiens) | 1.10 | 8.17 | 4.11E-03 |
| [COPI-independent Golgi-to-ER retrograde traffic](http://www.reactome.org/PathwayBrowser/#/R-HSA-6811436) | [46](http://www.pantherdb.org/tools/gxIdsList.do?acc=R-HSA-6811436&reflist=1) | [9](http://www.pantherdb.org/tools/gxIdsList.do?acc=R-HSA-6811436&list=Client%20Text%20Box%20Input&organism=Homo%20sapiens) | 1.10 | 8.17 | 4.11E-03 |
| [Kinesins](http://www.reactome.org/PathwayBrowser/#/R-HSA-983189) | [60](http://www.pantherdb.org/tools/gxIdsList.do?acc=R-HSA-983189&reflist=1) | [9](http://www.pantherdb.org/tools/gxIdsList.do?acc=R-HSA-983189&list=Client%20Text%20Box%20Input&organism=Homo%20sapiens) | 1.44 | 6.27 | 3.35E-02 |
| ↳ Hemostasis | [590](http://www.pantherdb.org/tools/gxIdsList.do?acc=R-HSA-109582&reflist=1) | [32](http://www.pantherdb.org/tools/gxIdsList.do?acc=R-HSA-109582&list=Client%20Text%20Box%20Input&organism=Homo%20sapiens) | 14.12 | 2.27 | 3.87E-02 |
| [The role of GTSE1 in G2/M progression after G2 checkpoint](http://www.reactome.org/PathwayBrowser/#/R-HSA-8852276) | [77](http://www.pantherdb.org/tools/gxIdsList.do?acc=R-HSA-8852276&reflist=1) | [10](http://www.pantherdb.org/tools/gxIdsList.do?acc=R-HSA-8852276&list=Client%20Text%20Box%20Input&organism=Homo%20sapiens) | 1.84 | 5.43 | 3.95E-02 |
| [COPI-mediated anterograde transport](http://www.reactome.org/PathwayBrowser/#/R-HSA-6807878) | [95](http://www.pantherdb.org/tools/gxIdsList.do?acc=R-HSA-6807878&reflist=1) | [11](http://www.pantherdb.org/tools/gxIdsList.do?acc=R-HSA-6807878&list=Client%20Text%20Box%20Input&organism=Homo%20sapiens) | 2.27 | 4.84 | 4.41E-02 |
| ↳ Transport to the Golgi and subsequent modification | [179](http://www.pantherdb.org/tools/gxIdsList.do?acc=R-HSA-948021&reflist=1) | [16](http://www.pantherdb.org/tools/gxIdsList.do?acc=R-HSA-948021&list=Client%20Text%20Box%20Input&organism=Homo%20sapiens) | 4.28 | 3.73 | 1.76E-02 |
| [MHC class II antigen presentation](http://www.reactome.org/PathwayBrowser/#/R-HSA-2132295) | [119](http://www.pantherdb.org/tools/gxIdsList.do?acc=R-HSA-2132295&reflist=1) | [13](http://www.pantherdb.org/tools/gxIdsList.do?acc=R-HSA-2132295&list=Client%20Text%20Box%20Input&organism=Homo%20sapiens) | 2.85 | 4.56 | 1.52E-02 |
| Metabolism of lipids and lipoproteins | [712](http://www.pantherdb.org/tools/gxIdsList.do?acc=R-HSA-556833&reflist=1) | [41](http://www.pantherdb.org/tools/gxIdsList.do?acc=R-HSA-556833&list=Client%20Text%20Box%20Input&organism=Homo%20sapiens) | 17.04 | 2.41 | 6.04E-04 |
| ↳ Metabolism | [1972](http://www.pantherdb.org/tools/gxIdsList.do?acc=R-HSA-1430728&reflist=1) | [92](http://www.pantherdb.org/tools/gxIdsList.do?acc=R-HSA-1430728&list=Client%20Text%20Box%20Input&organism=Homo%20sapiens) | 47.20 | 1.95 | 9.42E-07 |

**Downregulated by eLDL loading (HG>6)**

|  | [**#**](http://www.pantherdb.org/tools/compareToRefList.jsp?sortOrder=2&sortList=Homo%20sapiens) **total** | [**#**](http://www.pantherdb.org/tools/compareToRefList.jsp?sortOrder=2&sortList=Client%20Text%20Box%20Input&sortField=num) **found** | [**expected**](http://www.pantherdb.org/tools/compareToRefList.jsp?sortOrder=2&sortList=Client%20Text%20Box%20Input&sortField=exp) | [**Fold Enrichment**](http://www.pantherdb.org/tools/compareToRefList.jsp?sortOrder=2&sortList=Client%20Text%20Box%20Input&sortField=foldEnrich) | [**P value**](http://www.pantherdb.org/tools/compareToRefList.jsp?sortOrder=1&sortList=Client%20Text%20Box%20Input&sortField=pval) |
| --- | --- | --- | --- | --- | --- |
| [Cholesterol biosynthesis via lathosterol](http://www.reactome.org/PathwayBrowser/#/R-HSA-6807062) | [4](http://www.pantherdb.org/tools/gxIdsList.do?acc=R-HSA-6807062&reflist=1) | [4](http://www.pantherdb.org/tools/gxIdsList.do?acc=R-HSA-6807062&list=Client%20Text%20Box%20Input&organism=Homo%20sapiens) | .14 | 28.57 | 2.52E-02 |
| ↳ [Cholesterol biosynthesis](http://www.reactome.org/PathwayBrowser/#/R-HSA-191273) | [23](http://www.pantherdb.org/tools/gxIdsList.do?acc=R-HSA-191273&reflist=1) | [15](http://www.pantherdb.org/tools/gxIdsList.do?acc=R-HSA-191273&list=Client%20Text%20Box%20Input&organism=Homo%20sapiens) | .80 | 18.63 | 2.17E-11 |
| ↳ [Metabolism of lipids and lipoproteins](http://www.reactome.org/PathwayBrowser/#/R-HSA-556833) | [712](http://www.pantherdb.org/tools/gxIdsList.do?acc=R-HSA-556833&reflist=1) | [53](http://www.pantherdb.org/tools/gxIdsList.do?acc=R-HSA-556833&list=Client%20Text%20Box%20Input&organism=Homo%20sapiens) | 24.92 | 2.13 | 6.81E-04 |
| ↳ [Metabolism](http://www.reactome.org/PathwayBrowser/#/R-HSA-1430728) | [1972](http://www.pantherdb.org/tools/gxIdsList.do?acc=R-HSA-1430728&reflist=1) | [106](http://www.pantherdb.org/tools/gxIdsList.do?acc=R-HSA-1430728&list=Client%20Text%20Box%20Input&organism=Homo%20sapiens) | 69.02 | 1.54 | 1.33E-02 |
| [Cholesterol biosynthesis via desmosterol](http://www.reactome.org/PathwayBrowser/#/R-HSA-6807047) | [4](http://www.pantherdb.org/tools/gxIdsList.do?acc=R-HSA-6807047&reflist=1) | [4](http://www.pantherdb.org/tools/gxIdsList.do?acc=R-HSA-6807047&list=Client%20Text%20Box%20Input&organism=Homo%20sapiens) | .14 | 28.57 | 2.52E-02 |
| [Metallothioneins bind metals](http://www.reactome.org/PathwayBrowser/#/R-HSA-5661231) | [11](http://www.pantherdb.org/tools/gxIdsList.do?acc=R-HSA-5661231&reflist=1) | [6](http://www.pantherdb.org/tools/gxIdsList.do?acc=R-HSA-5661231&list=Client%20Text%20Box%20Input&organism=Homo%20sapiens) | .38 | 15.58 | 5.68E-03 |
| ↳ [Response to metal ions](http://www.reactome.org/PathwayBrowser/#/R-HSA-5660526) | [11](http://www.pantherdb.org/tools/gxIdsList.do?acc=R-HSA-5660526&reflist=1) | [6](http://www.pantherdb.org/tools/gxIdsList.do?acc=R-HSA-5660526&list=Client%20Text%20Box%20Input&organism=Homo%20sapiens) | .38 | 15.58 | 5.68E-03 |
| [Activation of gene expression by SREBF (SREBP)](http://www.reactome.org/PathwayBrowser/#/R-HSA-2426168) | [40](http://www.pantherdb.org/tools/gxIdsList.do?acc=R-HSA-2426168&reflist=1) | [17](http://www.pantherdb.org/tools/gxIdsList.do?acc=R-HSA-2426168&list=Client%20Text%20Box%20Input&organism=Homo%20sapiens) | 1.40 | 12.14 | 3.47E-10 |
| ↳ [Regulation of cholesterol biosynthesis by SREBP (SREBF)](http://www.reactome.org/PathwayBrowser/#/R-HSA-1655829) | [53](http://www.pantherdb.org/tools/gxIdsList.do?acc=R-HSA-1655829&reflist=1) | [18](http://www.pantherdb.org/tools/gxIdsList.do?acc=R-HSA-1655829&list=Client%20Text%20Box%20Input&organism=Homo%20sapiens) | 1.85 | 9.70 | 2.74E-09 |
| [Innate Immune System](http://www.reactome.org/PathwayBrowser/#/R-HSA-168249) | [790](http://www.pantherdb.org/tools/gxIdsList.do?acc=R-HSA-168249&reflist=1) | [52](http://www.pantherdb.org/tools/gxIdsList.do?acc=R-HSA-168249&list=Client%20Text%20Box%20Input&organism=Homo%20sapiens) | 27.65 | 1.88 | 2.69E-02 |
| ↳ [Immune System](http://www.reactome.org/PathwayBrowser/#/R-HSA-168256) | [1604](http://www.pantherdb.org/tools/gxIdsList.do?acc=R-HSA-168256&reflist=1) | [98](http://www.pantherdb.org/tools/gxIdsList.do?acc=R-HSA-168256&list=Client%20Text%20Box%20Input&organism=Homo%20sapiens) | 56.14 | 1.75 | 1.27E-04 |

**Upregulated by oxLDL loading (HG>6)**

|  | [**#**](http://www.pantherdb.org/tools/compareToRefList.jsp?sortOrder=2&sortList=Homo%20sapiens) **total** | [**#**](http://www.pantherdb.org/tools/compareToRefList.jsp?sortOrder=2&sortList=Client%20Text%20Box%20Input&sortField=num) **found** | [**expected**](http://www.pantherdb.org/tools/compareToRefList.jsp?sortOrder=2&sortList=Client%20Text%20Box%20Input&sortField=exp) | [**Fold Enrichment**](http://www.pantherdb.org/tools/compareToRefList.jsp?sortOrder=2&sortList=Client%20Text%20Box%20Input&sortField=foldEnrich) | [**P value**](http://www.pantherdb.org/tools/compareToRefList.jsp?sortOrder=1&sortList=Client%20Text%20Box%20Input&sortField=pval) |
| --- | --- | --- | --- | --- | --- |
| Metallothioneins bind metals | 11 | 8 | .32 | 24.72 | 3.81E-06 |
| ↳ Response to metal ions | 11 | 8 | .32 | 24.72 | 3.81E-06 |
| ↳ [Metabolism](http://www.reactome.org/PathwayBrowser/#/R-HSA-1430728) | 1972 | 116 | 58.02 | 2.00 | 1.09E-09 |
| Fatty acid, triacylglycerol, and ketone body metabolism | 225 | 21 | 6.62 | 3.17 | 9.65E-03 |
| ↳ Metabolism of lipids and lipoproteins | 712 | 50 | 20.95 | 2.39 | 4.39E-05 |

**Downregulated by oxLDL loading (HG>6)**

|  | [**#**](http://www.pantherdb.org/tools/compareToRefList.jsp?sortOrder=2&sortList=Homo%20sapiens) **total** | [**#**](http://www.pantherdb.org/tools/compareToRefList.jsp?sortOrder=2&sortList=Client%20Text%20Box%20Input&sortField=num) **found** | **expected** | **Fold Enrichment** | **P value** |
| --- | --- | --- | --- | --- | --- |
| Cholesterol biosynthesis via lathosterol | 4 | 4 | .15 | 26.05 | 3.61E-02 |
| ↳ Cholesterol biosynthesis | 23 | 16 | .88 | 18.12 | 4.41E-12 |
| ↳ Metabolism of lipids and lipoproteins | 712 | 53 | 27.33 | 1.94 | 1.01E-02 |
| Cholesterol biosynthesis via desmosterol | 4 | 4 | .15 | 26.05 | 3.61E-02 |
| Activation of gene expression by SREBF (SREBP) | 40 | 12 | 1.54 | 7.82 | 1.46E-04 |
| ↳ Regulation of cholesterol biosynthesis by SREBP (SREBF) | 53 | [13](http://www.pantherdb.org/tools/gxIdsList.do?acc=R-HSA-1655829&list=Client%20Text%20Box%20Input&organism=Homo%20sapiens) | 2.03 | 6.39 | 4.15E-04 |
| Chemokine receptors bind chemokines | 57 | 12 | 2.19 | 5.48 | 5.67E-03 |
| ↳ Class A/1 (Rhodopsin-like receptors) | 323 | 33 | 12.40 | 2.66 | 1.22E-03 |
| ↳ GPCR ligand binding | 449 | 38 | 17.23 | 2.20 | 1.44E-02 |
| ↳ Signal Transduction | 2460 | 151 | 94.43 | 1.60 | 8.83E-06 |
| Interferon gamma signaling | 91 | 15 | 3.49 | 4.29 | 6.84E-03 |
| ↳ Interferon Signaling | 192 | 23 | 7.37 | 3.12 | 4.78E-03 |
| ↳ Cytokine Signaling in Immune system | 610 | 56 | 23.41 | 2.39 | 7.08E-06 |
| ↳ Immune System | 1604 | 138 | 61.57 | 2.24 | 1.24E-15 |
| Immunoregulatory interactions between a Lymphoid and a non-Lymphoid cell | 156 | 19 | 5.99 | 3.17 | 2.75E-02 |
| ↳ Adaptive Immune System | 821 | 64 | 31.51 | 2.03 | 2.22E-04 |
| Platelet activation, signaling and aggregation | 275 | 29 | 10.56 | 2.75 | 3.11E-03 |
| ↳ Hemostasis | 590 | 61 | 22.65 | 2.69 | 1.41E-08 |
| Signaling by Rho GTPases | 383 | 38 | 14.70 | 2.58 | 3.63E-04 |
| Gastrin-CREB signaling pathway via PKC and MAPK | 421 | 36 | 16.16 | 2.23 | 1.99E-02 |
| Cell Cycle, Mitotic | 471 | 39 | 18.08 | 2.16 | 1.79E-02 |
| Innate Immune System | 790 | 62 | 30.32 | 2.04 | 2.81E-04 |

**Upregulated eLDL after deloading (HG>6)**

|  | [**#**](http://www.pantherdb.org/tools/compareToRefList.jsp?sortOrder=2&sortList=Homo%20sapiens) **total** | [**#**](http://www.pantherdb.org/tools/compareToRefList.jsp?sortOrder=2&sortList=Client%20Text%20Box%20Input&sortField=num) **found** | **expected** | **Fold Enrichment** | **P value** |
| --- | --- | --- | --- | --- | --- |
| Cholesterol biosynthesis | 23 | 8 | .39 | 20.61 | 1.50E-05 |
| ↳ Metabolism | 1972 | 60 | 33.29 | 1.80 | 1.11E-02 |
| Glutathione conjugation | 38 | 7 | .64 | 10.91 | 8.57E-03 |

**Downregulated eLDL after deloading (HG>6)**

|  | [**#**](http://www.pantherdb.org/tools/compareToRefList.jsp?sortOrder=2&sortList=Homo%20sapiens) **total** | [**#**](http://www.pantherdb.org/tools/compareToRefList.jsp?sortOrder=2&sortList=Client%20Text%20Box%20Input&sortField=num) **found** | **expected** | **Fold Enrichment** | **P value** |
| --- | --- | --- | --- | --- | --- |
| Chemokine receptors bind chemokines | 57 | 8 | 1.03 | 7.79 | 2.08E-02 |

**Upregulated oxLDL after deloading (HG>6)**

|  | **#** | **#** | **expected** | **Fold Enrichment** | **P value** |
| --- | --- | --- | --- | --- | --- |
| Cholesterol biosynthesis via lathosterol | 4 | 4 | .16 | 24.73 | 4.42E-02 |
| ↳ Cholesterol biosynthesis | 23 | 19 | .93 | 20.43 | 1.27E-15 |
| ↳ Metabolism of lipids and lipoproteins | 712 | 62 | 28.79 | 2.15 | 4.87E-05 |
| Cholesterol biosynthesis via desmosterol | 4 | 4 | .16 | 24.73 | 4.42E-02 |
| Microtubule-dependent trafficking of connexons from Golgi to the plasma membrane | 18 | 12 | .73 | 16.49 | 3.91E-08 |
| ↳ Transport of connexons to the plasma membrane | 19 | 12 | .77 | 15.62 | 7.21E-08 |
| ↳ Gap junction assembly | 36 | 12 | 1.46 | 8.24 | 8.28E-05 |
| ↳ Gap junction trafficking | 47 | 16 | 1.90 | 8.42 | 3.70E-07 |
| ↳ Gap junction trafficking and regulation | 48 | 16 | 1.94 | 8.24 | 4.99E-07 |
| Post-chaperonin tubulin folding pathway | 22 | 12 | .89 | 13.49 | 3.75E-07 |
| ↳ Protein folding | 100 | 17 | 4.04 | 4.20 | 2.11E-03 |
| Formation of tubulin folding intermediates by CCT/TriC | 25 | 13 | 1.01 | 12.86 | 1.19E-07 |
| ↳ Cooperation of Prefoldin and TriC/CCT in actin and tubulin folding | 32 | 14 | 1.29 | 10.82 | 2.06E-07 |
| ↳ Chaperonin-mediated protein folding | 94 | 17 | 3.80 | 4.47 | 9.21E-04 |
| RHO GTPases activate IQGAPs | 30 | 15 | 1.21 | 12.37 | 7.12E-09 |
| ↳ RHO GTPase Effectors | 269 | 27 | 10.88 | 2.48 | 4.13E-02 |
| ↳ Signaling by Rho GTPases | 383 | 37 | 15.49 | 2.39 | 3.28E-03 |
| ↳ Signal Transduction | 2460 | 140 | 99.47 | 1.41 | 4.13E-02 |
| Recruitment of NuMA to mitotic centrosomes | 28 | 12 | 1.13 | 10.60 | 5.44E-06 |
| Prefoldin mediated transfer of substrate to CCT/TriC | 27 | 11 | 1.09 | 10.08 | 4.09E-05 |
| Activation of gene expression by SREBF (SREBP) | 40 | 15 | 1.62 | 9.27 | 3.68E-07 |
| ↳ Regulation of cholesterol biosynthesis by SREBP (SREBF) | 53 | 16 | 2.14 | 7.47 | 2.02E-06 |
| Recycling pathway of L1 | 46 | 16 | 1.86 | 8.60 | 2.72E-07 |
| ↳ L1CAM interactions | 115 | 18 | 4.65 | 3.87 | 3.26E-03 |
| ↳ Axon guidance | 546 | 52 | 22.08 | 2.36 | 4.31E-05 |
| ↳ Developmental Biology | 806 | 63 | 32.59 | 1.93 | 1.50E-03 |
| COPI-independent Golgi-to-ER retrograde traffic | 46 | 13 | 1.86 | 6.99 | 1.52E-04 |
| Translocation of GLUT4 to the plasma membrane | 79 | 18 | 3.19 | 5.63 | 1.45E-05 |
| Intraflagellar transport | 53 | 12 | 2.14 | 5.60 | 4.62E-03 |
| Kinesins | 60 | 13 | 2.43 | 5.36 | 2.88E-03 |
| ↳ Factors involved in megakaryocyte development and platelet production | 159 | 24 | 6.43 | 3.73 | 1.29E-04 |
| ↳ Hemostasis | 590 | 60 | 23.86 | 2.52 | 3.11E-07 |
| The role of GTSE1 in G2/M progression after G2 checkpoint | 77 | 13 | 3.11 | 4.18 | 3.97E-02 |
| COPI-mediated anterograde transport | 95 | 15 | 3.84 | 3.90 | 2.08E-02 |
| RHO GTPases Activate Formins | 127 | 18 | 5.14 | 3.51 | 1.25E-02 |
| Cytokine Signaling in Immune system | 610 | 49 | 24.67 | 1.99 | 1.25E-02 |
| ↳ Immune System | 1604 | 125 | 64.86 | 1.93 | 3.84E-09 |
| Innate Immune System | 790 | 60 | 31.94 | 1.88 | 6.64E-03 |

**Downregulated oxLDL after deloading (HG>6)**

|  | **#** | **#** | **expected** | **Fold Enrichment** | **P value** |
| --- | --- | --- | --- | --- | --- |
| Metallothioneins bind metals | 11 | 9 | .45 | 19.86 | 2.53E-06 |
| ↳ Response to metal ions | 11 | 9 | .45 | 19.86 | 2.53E-06 |
| Cytosolic tRNA aminoacylation | 24 | 10 | .99 | 10.11 | 1.71E-04 |
| ↳ tRNA Aminoacylation | 42 | 10 | 1.73 | 5.78 | 2.38E-02 |
| Kinesins | 60 | 12 | 2.47 | 4.85 | 1.91E-02 |
| Resolution of Sister Chromatid Cohesion | 114 | 21 | 4.70 | 4.47 | 4.44E-05 |
| ↳ Mitotic Prometaphase | 122 | 23 | 5.03 | 4.58 | 6.37E-06 |
| ↳ M Phase | 281 | 31 | 11.58 | 2.68 | 2.37E-03 |
| ↳ Cell Cycle, Mitotic | 471 | 45 | 19.40 | 2.32 | 5.82E-04 |
| ↳Cell Cycle | 575 | 51 | 23.69 | 2.15 | 8.67E-04 |
| Separation of Sister Chromatids | 178 | 22 | 7.33 | 3.00 | 1.45E-02 |
| ↳ Mitotic Anaphase | 186 | 22 | 7.66 | 2.87 | 2.81E-02 |
| ↳ Mitotic Metaphase and Anaphase | 187 | 22 | 7.70 | 2.86 | 3.04E-02 |
